# Efficacy of Antiseptic Solutions in Treatment of *Staphylococcus Aureus* Infected Surgical Wounds with Patches of Vascular Graft: An Experimental Study in Rats

**DOI:** 10.3390/medicina55040106

**Published:** 2019-04-15

**Authors:** Elvyra Stanevičiūtė, Inga Urtė Builytė, Martynas Ridziauskas, Justinas Besusparis, Agnė Kirkliauskienė, Vaidotas Zabulis, Linas Davainis, Gabrielė Valiūnaitė, Vytautas Triponis, Vytautas Sirvydis

**Affiliations:** 1 Vilnius University Faculty of Medicine, Vilnius, LT-03101, Lithuania; u.builyte@gmail.com (I.U.B.); martynas.ridziauskas@gmail.com (M.R.); justinas.besusparis@gmail.com (J.B.); agne.kirkliauskiene@mf.vu.lt (A.K.); linasdaw@gmail.com (L.D.); g.valiunaite@gmail.com (G.V.); vytautas.triponis@mf.vu.lt (V.T.); vytautas.sirvydis@santa.lt (V.S.); 2 National Center of Pathology, affiliate of Vilnius University Hospital Santaros Klinikos, Vilnius, LT-08406, Lithuania; 3 Vilnius University Hospital Santaros Klinikos, Vilnius, LT-08661, Lithuania; vaidas.zabulis@gmail.com

**Keywords:** rat model, vascular graft infection, *S. aureus*, antiseptic

## Abstract

*Background and objectives:* Treatment of a prosthetic vascular graft infection (PVGI) remains a challenging problem in vascular surgery. The aim of this study was to design a novel rat model for treatment of peripheral vascular prosthesis infection caused by *Staphylococcus aureus (S. aureus)* and to determine the efficacy of different antiseptic solutions in suppressing or eradicating infection from the wound and the graft material itself. *Materials and methods:* A piece of Dacron vascular prosthesis was surgically implanted at the dorsum of 48 Wistar rats and the wounds were infected with 5 McFarland standard inoculum of *S. aureus*. Suppurating wounds were daily irrigated with different antiseptic solutions: octenidine dihydrochloride, povidone-iodine, chlorhexidine digluconate, and sterile saline. The antimicrobial action of antiseptics was defined according to their capability to eradicate bacteria from the graft surroundings and bacteriological examination of the graft itself. Extended studies on wound microbiology, cytology, and histopathology were performed with an additional group of 10 rats, treated with the most effective antiseptic-octenidine dihydrochloride. *Results:* Four-day treatment course with octenidine, povidone-iodine, and chlorhexidine resulted in 99.98% (*p* = 0.0005), 90.73% (*p* = 0.002), and 65.97% (*p* = 0.004) decrease in *S. aureus* colonies in wound washouts, respectively. The number of *S. aureus* colonies increased insignificantly by 19.72% (*p* = 0.765) in control group. Seven-day treatment course with octenidine eradicated viable bacteria from nine out of 10 wound washouts and sterilized one vascular graft. *Conclusions*: A reproducible rat model of PVGI with a thriving *S. aureus* infection was designed. It is a first PVGI animal model where different antiseptic solutions were applied as daily irrigations to treat peripheral PVGI. Seven-day treatment with octenidine eradicated bacteria from the wound washouts for 90% of rats and one vascular graft. Further studies are needed to investigate if irrigations with octenidine could properly cure vascular bed from infection to assure a successful implantation of a new synthetic vascular substitute.

## 1. Introduction

Prosthetic vascular graft infection (PVGI) is an uncommon, but potentially life-threatening complication in vascular surgery. Although the rate of PVGIs has been reported to range from 1 to 6% in different vascular surgery clinics [1,2], association with limb amputation rates from 5 to 70% and overwhelming mortality rates (20–75%) keeps this vascular complication at the top of a vascular surgeon’s concern list [1,3,4].

PVGI may develop perioperatively, due to breaches in asepsis, such as poor skin’s disinfection, or postoperatively, mostly due to surgical site infection or systemic bacteremia. Once PVGI has been diagnosed, there are several management strategies, but no widely-accepted guidelines on the best treatment approach. Traditionally, aggressive surgical debridement of infected tissues together with graft excision, extra-anatomic revascularization, and high doses of systemic antibiotics are recommended [1]. However, this approach is not always suitable, especially when aorta is involved, when a patient suffers from serious comorbidities and would not survive an aggressive treatment approach, or the infectious agent is multi-drug resistant. Not many antibiotics can penetrate biofilms that are formed by *Staphylococci* [5], the most common causative agent of early and late onset PVGIs [4,6,7,8,9]. Therefore, several conservative treatment strategies are being developed and found to be successful in certain PVGI cases [10,11]. For the most part, conservative treatment options are used for treatment of peripheral vascular graft infections in the extremities and include negative pressure wound therapy and local wound drainage with constant or repetitive irrigations [11]. The wound irrigations may be performed using sterile saline solution [12], antibiotic, or antiseptic solutions. Although antiseptics are usually used to prevent a surgical site infection, they are becoming increasingly used for treatment of various infections. In the era of rapidly developing bacterial resistance, antiseptics are superior to antibiotics due to their minimal development of bacterial resistance [13]. To investigate a possible conservative local PVGI treatment method—irrigation of a wound with different antiseptic solutions, we designed an animal (rat) model with a *Staphylococcus aureus (S. aureus)* infected patch of vascular graft and treated the wound with repetitive irrigations. Our aims were to test if irrigation with antiseptics is an effective treatment method to eradicate bacteria from the infected vascular graft material and the surrounding tissues and compare the efficacy of different, commonly used antiseptic solutions. Analogous study on treatment of rats’ PVGI with local irrigations of antiseptics was not found to be published previously, since most studies published on prophylaxis or treatment of animal PVGI describe using local or systemic antibiotic therapy.

## 2. Materials and Methods

A new rat model for conservative treatment of PVGI was designed. All investigators have received FELASA C category diplomas prior to the experiment. Permission to perform an experimental study was granted by Lithuanian State Food and Veterinary Service (permission no. G2–43). Animals for the experiment—white Wistar rats of male and female genders—were obtained from Vilnius University Life Sciences Center. All rats were 3–6 months of age and 239–450 g weight. Several pilot studies were carried out to estimate a proper *S. aureus* dose and time period to cause a suppurative incisional wound infection with implanted vascular graft for rats that would persist for at least two weeks. Six rats, which were used in the pilot studies, were not included in the further experiments. The main study was performed in two stages. The first stage was carried out to test a newly created rat model for treatment of PVGI caused by *S. aureus* and to evaluate the efficacy of three different antiseptic solutions. The second stage of the experiment was performed with the most effective antiseptic solution for an extended treatment period. The repeatability of a newly created rat model was tested and microbiological and cytological wound findings were evaluated. Wound tissues were investigated histopathologically.

### 2.1. Infectious Agent

*S. aureus* strain no. 215, isolated from a human clinical specimen and stored in Vilnius University Faculty of Medicine Department of Physiology, Biochemistry, Microbiology, and Laboratory Medicine, was used as an infectious agent. The selected strain was a Panton-Valentin leucocidine (PVL) positive biofilm-producer, resistant to tetracyclin and penicillin. The culture was cultivated on 5% sheep blood agar (“Bio-Rad”, France) at 35 °C for 24 h, then added to sterile 0.85% sodium chloride solution, vortexed, and evaluated with McFarland densitometer. For the pilot study, three different suspensions were prepared, equivalent to 1, 3, and 5 McFarland standard. In total, 1 mL of each suspension was added to incisional wound at the dorsum of six rats (two rats for each suspension) with a vascular graft in it, the wound was closed and observed for 1 week (detailed methodology described in the section *Testing the efficacy of antiseptics in treatment of rat PVGI*). The amount of 1 mL of *S. aureus* suspension, equivalent to 5 McFarland standard and five days postoperatively, proved to be adequate to confirm a flourishing PVGI for a rat, both clinically and microbiologically.

### 2.2. Testing the Efficacy of Antiseptics in Treatment of Rat PVGI

Each rodent (n = 48) was caged separately a week before the surgery to get accustomed to the new surroundings. The balanced standardized pellet diet, water, and same controlled conditions were ensured. Preoperatively, all rats were weighed and their rectal temperature was measured. The weight and rectal temperatures of rats were again evaluated at the peak of the suppuration before starting the treatment (day six), daily before performing irrigations with antiseptic solutions (day six, seven, eight, and nine) and at the end of the experiment (day 10). Anesthesia was induced using 10% ketamine 40 mg/kg (“Bio-ketan”, Vetoquinol Biowet Sp. Zoo, Poland) and 2% xylazin 5 mg/kg (“Xylazin”, Bela-Pharm GmbH and Co. KG, Germany), given by intraperitoneal injection. Operating table, instruments, surgical clothing, and surgical site were prepared following aseptic and antiseptic principles. For each rodent, hair from the surgical site was shaved with electric clippers and skin was disinfected with skin antiseptic (“Cutasept F”, Bode Chemie Hamburg, Germany). A sterile non-impregnated woven 6 mm diameter Dacron graft (Twillweave^®^, Vascutek Terumo) was cut into even pieces (9.4 mm × 6 mm). For each rat, a-two-centimeter-long paravertebral cutaneous and subcutaneous incision was made on the right side of the spine. Subcutaneous tissue was bluntly dissected from the muscular fascia to create a recess for implantation of a vascular graft. Wounds with implants were inoculated with 1 mL of *S. aureus* suspension, equivalent to 5 McFarland standard, using a sterile automatic volume pipette right before the last suture knots inside (with 3-0 monofilament (Ethicon, Johnson and Johnson)) was closed. All rats were kept in separate cages for five days in the same controlled conditions and received standardized food.

On day six of the experiment (time determined during the pilot study) samples from the wounds were taken by disinfecting the skin, injecting 1 mL of sterile saline solution with a syringe into the wound, and drawing the sample out. The washouts were then serially diluted (10-fold dilution assay) in 96-well microplates using phosphate buffer solution (PBS) as a diluent and plated on Mannitol-salt agar (Liofilchem, Italy) for enumeration of viable *S. aureus* colony forming units (CFUs). The agar plates were incubated at 35 °C for 24 h and then evaluated. The bacteriological samples from the nidus were taken again at day eight (before daily irrigation) and 10 (last irrigation performed on day nine) of the experiment to monitor bacterial growth dynamics.

Immediately after samples were taken on day six, the rodents were randomly divided into four groups of 12 without knowing their degree of suppuration. Daily irrigations with different antiseptics (0.1% octenidine dihydrochloride (Octenisept^®^, Schülke and Mayr GmbH, Germany), 10% povidone-iodine (Betadine^®^, EGIS Pharmaceuticals LTD, Hungary), and 0.02% chlorhexidine digluconate solution (Fresenius Kabi AG, Germany)) or sterile saline (control group) were started on day six by injecting 1 mL of antiseptic into the wound cavity, keeping it for 5 min and aspirating it with a syringe. This treatment was carried out daily for four days.

Experimental animals were monitored every day after the operation. It included behavior, weight, rectal temperatures, food, and water intake. The experiment was terminated at day 10. All laboratory animals were euthanized in the CO_2_ gas chamber. After euthanizing the animals, vascular grafts were aseptically removed from the wounds, placed into sterile test tubes with 5 mL of PBS, and vortexed for one hour at 500 rpm to detach the bacterial remains from the grafts. The suspensions were serially diluted (10-fold dilution assay) and plated on Mannitol-salt agar plates. After incubating the plates at 35 °C for 24 h, *S. aureus* CFUs were enumerated.

Some rats had to be excluded from the final analysis, described in detail in the Results section *Final sample size*.

### 2.3. Further Studies with the Most Effective Antiseptic for Rat PVGI

In the second series of experiments, only the most effective antiseptic, found in our previous study, was tested. Using the same methodology, a group of 10 rats was treated with octenidine dihydrochloride for an extended period of time; seven daily washings were performed. Bacteriological samples (wound washouts) were taken at days 6, 8, 10, 12, and 13 of the experiment. For cytological evaluation, 10 µL of not diluted washouts were taken from each sample and spread on clean glass slides, fixed with 10% buffered formalin, Gram-stained and scanned with ScanScope XT Slide Scanner (Leica Aperio Technologies, Vista, CA, USA). Digitalized slides were analyzed by using Halo software in two steps. Firstly, the classifier module was calibrated to recognize cellular areas from artefacts and background (glass). Then Multiplex IHC v1.2 mode was used to quantify inflammatory cells in areas that were previously identified as containing only cells. For bacteriological evaluation, the remaining washouts were serially diluted and plated on Mannitol-salt agar plates for enumeration of viable *S. aureus* CFUs (detailed methodology described in the section *Testing the efficacy of antiseptics in treatment of rat PVGI*).

The experiment was terminated at day 13 when the bacterial count in the wound washouts was completely eradicated for nine out of 10 rodents. Vascular grafts were removed and investigated as described in the section *Testing the efficacy of antiseptics in treatment of rat PVGI*. Blood samples were taken via cardiac puncture with sterile syringes, plated on Mannitol-salt agar plates, incubated at 35 °C for 24 h, and evaluated. For each rodent, wound fistula with a scab and a piece of subcutaneous and muscular perigraft tissue from the dorsum were excised. Wound fistulas were divided in halves. The parts meant for bacteriological evaluation were weighed, minced, and placed separately in the test tubes with 5 mL of sterile PBS and vortexed for one hour at 500 rpm. Then, the suspension was serially diluted with PBS and plated on Mannitol-salt agar plates. After keeping the plates at 35 °C for 24 h, bacterial count was evaluated. The parts of the fistulas meant for histopathological evaluation and the pieces of subcutaneous and muscular tissue were placed separately into 10% buffered formalin solution, embedded in paraffin, sliced into 5 µm-thick slices, and stained with hematoxylin and eosin. Each slide was evaluated both manually by the pathologist and digitally by Halo artificial intelligence (AI) classifier.

Manually, each tissue section was observed under the microscope (Olympus BX 40 with 10×, 20× and 40× objective lenses). The type of inflammation in the tissue section was determined by qualitatively describing inflammatory cells (granulocytes, lymphocytes, or macrophages/histiocytes) in the tissue. The severity of the inflammation was quantified by objectively enumerating inflammatory cell numbers in the tissues (no inflammation, no polymorphonuclear (PMN) cells—grade 0; minor inflammation, only scattered PMN,—grade 1; mild inflammation, intermediate density of PMN—grade 2; severe inflammation, PMN cells are densely packed—grade 3). The areas of necrosis were defined as an irreversible injury of cells and tissues without cells, or containing non-vital cells without clearly defined nuclei. The cytoplasm in these cells was usually homogenous and acidophilic.

Digitally, each tissue slide was scanned with a ScanScope XT Slide Scanner and analyzed with Halo AI classifier. The digital image analysis platform was manually calibrated to classify scanned images into three groups: glass, stroma (fat, skin, connective, and muscular tissue), and tissue necrosis. The area of tissue necrosis, calculated digitally and expressed in percent, was used for further comparisons.

### 2.4. Statistical Analysis

The data was analyzed using T-test and one-way ANOVA where data was known to be normally distributed. Other data was analyzed using Mann-Whitney-U, Kruskal-Wallis-H, and Wilcoxon-T tests. Statistical significance was assigned when *p* values were less than 0.05.

## 3. Results

### 3.1. Final Sample Size

One rat, which was assigned to control group, did not survive the surgery (possible anesthetic overdose). Two rats assigned to povidone-iodine group, and one rat assigned to chlorhexidine group, did not reach adequate level of suppuration (evaluation based on microbiological sample taken from the wound at day six of the experiment before starting the treatment) and were excluded from further study. One rat, assigned to chlorhexidine group, died at day eight of the experiment. Loss of appetite, resulting in very little daily food and water intake, reduced physical activity, dramatic weight loss (from 355 g to 188 g), and pus around the eye area were observed, which distinguished this rat from other rodents.

The final analysis of the first stage experiment was performed with 12 rats in octenidine, 10 rats in both povidone-iodine and chlorhexidine groups, and 11 rats in control group (total number of rodents n = 43). In the second stage of the experiment, where only octenidine was tested, final sample size was 10 rats.

### 3.2. Macroscopic Findings

The fluctuations in average body temperature were between the normal range for rats (35.8–37.5 °C) and did not differ significantly between the treatment and control groups.

The mean body weight of all rats at baseline (day six) was 317.6 ± 48.1 g and varied from 239 to 450 g. The mean rats’ body weight in the treatment groups was 318.6 ± 40.1 g for octenidine, 323.1 ± 58.2 g for povidone-iodine, 312.5 ± 48.6 g for chlorhexidine, and 316.2 ± 51.9 g for control group (Table 1). An ANOVA test showed that there was no evidence of weight non-homogeneity between the groups (*p* = 0.970).

A gradual decrease in the body weight was noticed along the time of treatment. At day 10 of the experiment (termination day), the body mass of all rodents have significantly decreased compared to day one (paired sample t-test, *p* = 0.0004). Further investigation revealed that a significant drop in the body weight was noticed only in groups treated with antiseptics, but not in the control group (Table 1). The most significant body weight loss was registered in the group treated with chlorhexidine, with average loss in weight for a rodent being 36.1 g (paired sample t-test, *p* = 0.043).

When compared to the control group, a significant loss in body weight was registered in povidone-iodine and chlorhexidine groups (two sample t-test, *p* = 0.022 and 0.038, respectively), but not in octenidine group (two sample t-test, *p* = 0.097).

**Figure 1:**
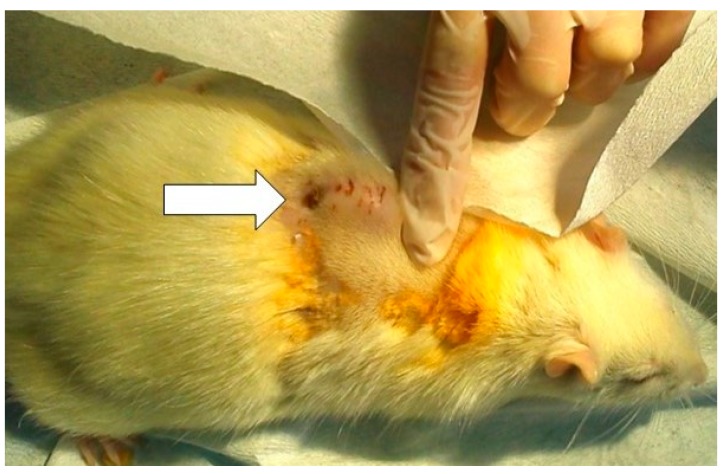
An arrow in the image points to a hardened swelling at the incision site with a purulent fistula on day six—before the beginning of treatment.

At day six, all rats were externally evaluated. All rodents had developed a hardened swelling at the incision site, but no erythema was noticed around the wounds (Figure 1). There were no suture leaks. Before the beginning of treatment, some of the rats had already formed a crust at the incision site. Gradually, all of the rats developed crusts at the site of incision and cutaneous fistulas with purulent discharge. The size of the scab around the incision site was not related to *S. aureus* colony counts from wound washouts or rat’s response to treatment.

### 3.3. Microbiological Findings

**Figure 2:**
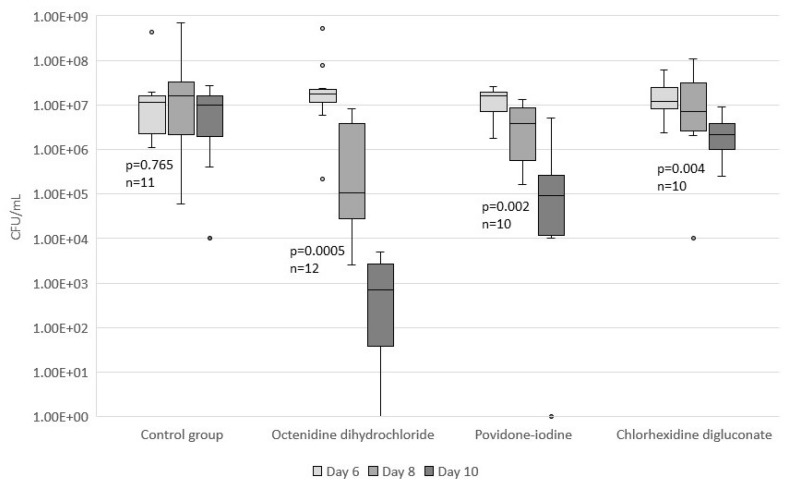
Changes in *S. aureus* colony counts in wound washouts along the time of treatment. Four-day treatment course resulted in significant drop in *S. aureus* colony counts for octenidine group (*p* = 0.0005, n = 12), povidone-iodine group (*p* = 0.002, n = 10), and chlorhexidine group (*p* = 0.004, n = 10). Insignificant change was detected in control group (*p* = 0.765, n = 11).

The changes in counts of *S. aureus* colonies in wound washouts on day six, eight, and 10 of the experiment were compared. Four-day treatment course (irrigations were performed on days six through nine of the experiment) with antiseptics resulted in CFU/mL median (interquartile range, IQR) change from 1.77 × 10^7^ (1.11x10^7^–2.28x10^7^) to 1.14 × 10^3^ (5.46 × 10^1^–2.96 × 10^3^), *p* = 0.0005 for octenidine, from 1.59 × 10^7^ (6.15 × 10^6^–2.02x10^7^) to 9.39 × 10^4^ (1.14 × 10^4^–3.08 × 10^5^), *p* = 0.002 for povidone-iodine and from 1.22 × 10^7^ (7.60 × 10^6^–2.95 × 10^7^) to 2.16 × 10^6^ (8.46 × 10^5^–4.02 × 10^6^), *p* = 0.004 for chlorhexidine groups. The change for the control group was from 1.13 × 10^7^ (2.29 × 10^6^–1.86 × 10^7^) to 9.70 × 10^6^ (1.55 × 10^6^–1.76x10^7^) and did not show statistical significance (*p* = 0.765). Compared to the control group, only the treatment with octenidine dihydrochloride statistically significantly lowered the counts of CFUs in the wounds (*p* = 0.019), though povidone-iodine also came close to the 5% statistical limit of significance (*p* = 0.061). The result of chlorhexidine treatment was not significant (*p* = 0.132) (Figure 2).

Complete eradication of viable *S. aureus* colonies from the surrounding tissues was confirmed for two rats treated with octenidine dihydrochloride, and one rat treated with povidone-iodine.

High counts (varying from 1.08 × 10^2^ to 8.05 × 10^7^ CFU/mL) of viable *S. aureus* colonies were found in microbiological samples from removed vascular grafts. These colony counts did not relate to *S. aureus* colony counts in samples from the wounds’ surrounding tissues.

### 3.4. Extended Studies with Octenidine Dihydrochloride

Additional series of analogous experiments were performed with another 10 rats treated with octenidine dihydrochloride. All rats survived the surgery. No trend or significant rectal body temperature changes were registered throughout the treatment course.

**Figure 3:**
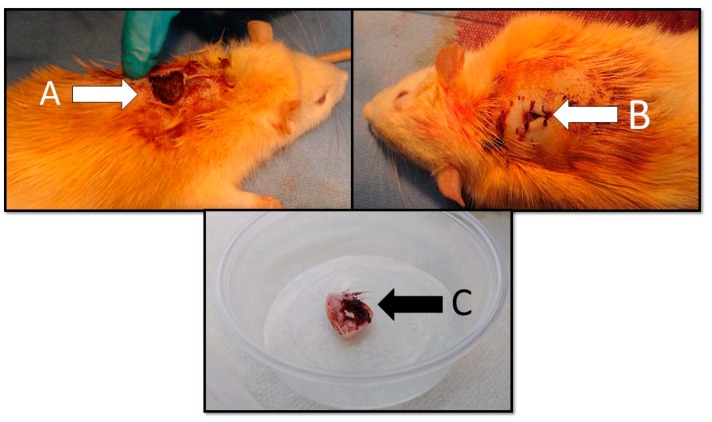
Macroscopic findings of the wound for rat treated with octenidine dihydrochloride (day 13): (**A**) a crust at the incision site; (**B**) a cutaneous fistula at the incision site; and (**C**) excised cutaneous fistula with a crust for microbiological and histopathological evaluation.

At day six, all rodents had developed hardened swelling at the incision site without erythema around the wound and no suture leaks. Most of the rats had formed a crust at the incision site. Two rats had developed fistulas with purulent discharge. The size of the crust around the incision site was not found to be related to *S. aureus* colony counts from wound washouts. From day 10, all rodents had scabs of varying sizes around the incision site and fistulas with purulent discharge (Figure 3). Massive excretion of pus through fistulas was observed at day 12 of the experiment for all rodents. Some of the crusts seemed a little bit detached from the skin, but none of them fell off completely.

A significant drop in body weight by 9.27% was registered throughout the experiment (paired sample t-test, *p* = 0.00005), with the body weight at day one being 267.3 ± 19.6 g and at day 13 being 242.2 ± 18.3 g.

**Figure 4:**
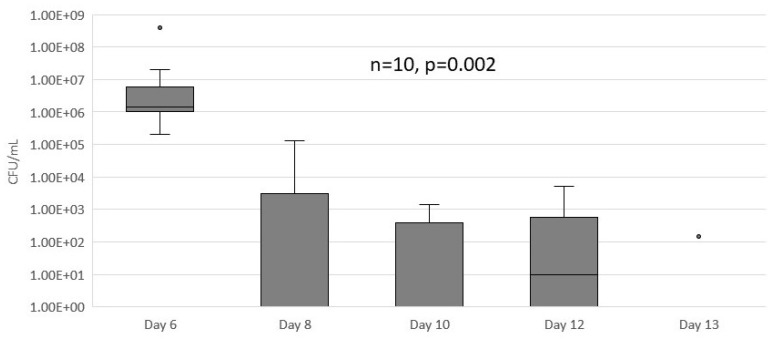
The results of extended PVGI treatment with octenidine dihydrochloride. Seven-day treatment course resulted in significant drop in *S. aureus* colony counts in wound washouts (*p* = 0.002, n = 10).

Seven-day-treatment course with octenidine dihydrochloride started by rodents having a median (IQR) of 1.5 × 10^6^ (1.0 × 10^6^–7.17 × 10^6^) CFU/mL in the wound samples. The treatment resulted in complete CFU eradication in all but one rodent, though it retained only 15 CFU/mL (Figure 4). The treatment results showed high statistical significance (Wilcoxon-T, *p* = 0.002).

After terminating the experiment at day 13, seven vascular grafts were removed from the rats. Another three vascular grafts were expelled from the wounds through the fistulas before terminating the experiment, and thus were not microbiologically evaluated. For one rat, no viable *S. aureus* colonies were found on the graft. In six other rats, vascular grafts colony counts varied from 20 CFU/mL to 2x10^7^ CFU/mL, with their median (IQR) being 8.0 × 10^1^ (2.3 × 10^1^–8.33 × 10^6^) CFU/mL.

Digital analysis of Gram-stained undiluted washouts from the wounds revealed that higher numbers of inflammatory cells at day 13 of the experiment significantly correlated with shorter *S. aureus* eradication times (the number of days taken for CFU/mL in wound washouts to decrease to zero) (Spearman’s Rho, r = −0.74, *p* = 0.013).

No dissemination of *S. aureus* into the bloodstream was detected.

Viable bacteria persisted in nine out of 10 fistulas. The median (IQR) of *S. aureus* colonies detected in each crust sample was 1.2 × 10^3^ (1.83 × 10^2^–8.95 × 10^5^) CFU/mL/g.

Histopathologically, the area of necrosis in fistula samples varied from 1.87% to 49.07%, with the median (IQR) being 17.33% (8–26%) without significant relation to rats’ levels of suppuration or response to treatment.

Two samples of fistulas presented with no inflammation (grade 0), two with minor PMN infiltration (grade 1), four with mild PMN infiltration (grade 2), and two with dense PMN infiltration (grade 3). The grade of inflammation was not found to correlate to the area of necrosis in the fistulas (Spearman’s Rho r = −0.07, *p* = 0.835).

The area of necrosis in the samples of subcutaneous and muscular tissues varied from 0 to 22.48%, with the median (IQR) being 1.5% (0–12%). According to the type and severity of inflammation, four rats were found to have developed xanthogranulomatous inflammation; three rats had mild, one had minor, and two had no PMN infiltration (Table 2).

## 4. Discussion

Despite constantly evolving antimicrobial therapies, PVGI still remains a challenge for a vascular surgeon, resulting in high numbers of limb amputations and death [1,2,3,14]. Both methicillin-susceptible and methicillin-resistant strains of *S. aureus*, responsible for a majority of early and late onset PVGIs, are increasingly developing resistance to antibiotics, such as cefazolin [3,15]. Another issue which makes it difficult to cope with *Staphylococcal* PVGI is bacteria’s ability to form biofilms on vascular prosthesis, as well as on other medical implants [16,17,18]. Considering these issues with antibiotics, we decided to investigate antiseptic solutions as a possible local treatment option for PVGI, as antiseptics are known to be able to penetrate biofilms and cause very little if any development of bacterial resistance.

*S. aureus* was chosen as the infection agent. Another aggravation in this study was woven Dacron graft, which was selected for the experiment. Polytetrafluoroethylene (PTFE) grafts were studied and found to be less susceptible to infection than Dacron grafts, most probably due to their even surface [19,20]. Yet, the structure of a woven Dacron graft, with its filaments arranged in an overlapping-pattern and forming an uneven, corrugated surface, enlarge the area of adhesion for bacterial cells and make them hardly accessible to antimicrobials and the host’s inflammatory cells.

The pilot studies carried out before the main experiment were crucial for designing a successful and reproducible animal model with surgical site infection (SSI). Choosing the right dose of inoculum is essential in animal models because deficient inoculum might be insufficient to cause a wound infection and result in wound colonization, triggering the host’s inflammatory response, and so promoting faster wound healing; however, high inoculum is associated with high mortality rates [21,22,23,24,25]. Differently from our experiment, Eliza et al. described 1 McFarland *S. aureus* inoculum to be sufficient to induce infection of the wound with 2 × 2 cm four different types of vascular graft for rats [26].

External appearance of the wounds in our experiment coincided mostly with those described in other animal models with SSI or PVGI. Since we created a closed wound and the abscesses formed subcutaneously, no redness around the edges of the sutured incision was observed, as described in animal models with open infected wounds [21]. Meulemans et al. also described swelling and nodules, as well as dry crusts at the inoculum sites, and only two rabbits out of 40 developed redness and skin rash [27].

A similar study by Eliza et al. was carried out for a period of four weeks and revealed that PVGI for rats caused by methicillin-susceptible *S. aureus* tended to heal on itself after day 14. Even though they used monofilament sutures to fix the pieces of grafts on the muscular plane, most of the grafts were expelled from the wound by the fourth week of the experiment. Although the dissemination of *Escherichia coli* was found in the bloodstream, *S. aureus* was not [26].

Zhao et al., in their study on chronic wounds, support our finding that despite bacteria’s eradication from the wounds, a great amount of viable bacteria remained localized in the scabs [28].

Analysis of the results of our experiment’s control group revealed that during the tested period rats did not recover on their own, because high counts of viable *S. aureus* colonies in wound washouts were found at the termination day of the experiment. Therefore, significant decrease of bacterial colonies in wound washouts during the course of treatment was a result of the treatment with antiseptics but not the mechanical irrigation or simply rats’ immune system.

Nevertheless, as discussed in literature, a positive CFU count alone does not confirm a wound infection. Without clinical signs of infection and inflammation, positive wound culture cannot differentiate wound infection from bacterial colonization, as its correlation with clinical symptoms of infection is poor [21,28,29,30]. Analysis of bacterial counts and abundance of inflammatory cells in wounds’ washouts as well as histopathological exam of wound fistulas and perigraft subcutaneous and muscular tissue with PMN infiltration of various extent clarified that we managed to cause an infection rather than bacterial colonization. According to cytological analysis of wound washouts, for rats with less aggressive immune response, viable *S. aureus* colony counts persisted for a longer period of time or were not eradicated at all until the end of treatment course.

Although treatment with octenidine dihydrochloride in our experiment resulted in 99–100% eradication of *S. aureus* colonies from the wound washouts, eradication of viable bacteria from the majority of grafts was not achieved. Using the same methodology, we achieved eradication of bacteria from the graft in one more rat treated with octenidine dihydrochloride [31]. To assure that viable bacteria were completely eradicated from the surrounding tissues, additional series of experiments need to be done to evaluate vascular graft bed tissues microbiologically.

Our study has several limitations. First, the sample size of our experiment was rather small and did not properly represent the ability of this method to sterilize the prosthesis. Increasing the sample size may verify our method’s efficacy in achieving eradication of bacteria from the grafts and confirm or deny our single case success. Also, the duration of the experiment was limited due to rats’ ability to heal rather quickly on their own. Additionally, an *in vivo* model with rodents cannot accurately represent a human clinical situation, and therefore assumptions about the method’s efficacy in treatment of human PVGI are vague and need clinical studies. However, our study demonstrated that irrigations with antiseptic solutions in the presence of an infected vascular graft are effective to reduce or even completely eradicate *S. aureus* colonies from the wounds. Clinically, this achievement is very significant for all patients with PVGI. For the ones who are physically fit enough to undergo a reconstructive surgery it could result in successful replacement of an infected graft with a new one, especially in peripheral artery disease. Likewise, for patients who are not fit for surgery, sterilizing a vascular graft bed could ensure graft preservation. Both options (preserving and substituting the graft) are widely discussed in literature as successful management options for PVGI. Most of the authors describe using antibiotic beads, negative pressure wound therapy, muscle-flap surgery, and irrigations with antibiotic [32,33], but not antiseptic solutions, which could become a cheap and easily accessible way to treat the condition.

## 5. Conclusions

A reproducible rat model of PVGI with a thriving *S. aureus* infection was designed. It is a first PVGI animal model where different antiseptic solutions were applied as daily irrigations to treat PVGI. Seven-day treatment with octenidine dihydrochoride eradicated bacteria from wound washouts for 90% of rats and one vascular graft. Further studies are needed to investigate if irrigations with octenidine could properly cure a vascular bed from infection to assure a successful implantation of a new synthetic vascular substitute.

## Figures and Tables

**Table 1:** Changes in rat’s body weight associated with antiseptic used for treatment of PVGI *.

Antiseptic Solution	Octenidine Dihydrochloride	Povidon-Iodine	Chlorhexidine Digluconate	Control Group (Sterile Saline)
Mean body weight at day 1, g	318.5 ± 40.1	323.1 ± 58.2	312.5 ± 48.6	316.2 ± 52.0
Mean body weight at day 10, g	299.3 ± 44.1	306.2 ± 60.6	276.4 ± 54.3	313.9 ± 50.0
Mean change of body weight, g	−19.25 ± 59.6	−16.9 ± 83.9	−36.1 ± 72.9	−2.3 ± 72.1
Effect size, Cohen’s d	−0.32	−0.2	−0.49	-0.03
Change in body weight, %	−5.9	−5.6	−11.5	−0.1
*p* value	0.048	0.004	0.043	0.566

* PVGI-prosthetic vascular graft infection

**Table 2:** Histopathological evaluation of subcutaneous and muscle tissue for rats treated with octenidine dihydrochloride.

Rat Number	Area of Necrosis in Subcutaneous and Muscle Tissue, %	Type of Inflammation in Subcutaneous and Muscle Tissue
1	1.90	Minor PMN *
2	0	Xantogranulomatous
3	11.58	Xantogranulomatous
4	0	Xantogranulomatous
5	1.17	None
6	20.77	Minor PMN
7	0	Xantogranulomatous
8	0	None
9	22.48	Mild PMN
10	10.04	Minor PMN

* PMN-polymorphonuclearic
